# Gross Abnormalities of the Appendix Vermiformis Noted in 3550 Autopsies

**Published:** 1906-03

**Authors:** A. P. Heineck

**Affiliations:** Chicago, Ill., Surgeon to Samaritan and Cook County Hospitals; Adjunct Professor of Surgery, Medical Department, University of Illinois; Professor of Surgery, Dearborn Medical College


### TEXAS MEDICAL JOURNAL.

ESTABLISHED JULY, 1885.

PUBLISHED MONTHLY.—SUBSCRIPTION $1.00 A YEAR.

VOL. XXI. AUSTIN, MARCH, 1906. No. 9.

The publisher is not responsible for the views of contributors.

For Texas Medical Journal.

### Gross Abnormalities of the Appendix Vermiformis Noted in 3550 Autopsies.

BY A. P. HEINECK, M. D., CHICAGO, ILL.,

Surgeon to Samaritan and Cook County Hospitals; Adjunct Professor of Surgery, Medical Department, University of Illinois; Professor of Surgery, Dearborn Medical College.

Anatomical, pathological and clinical data concerning the appendix vermiformis are always of a practical interest to the medical practitioner. The frequency of pathological conditions in this organ, be they of a degenerative, of an inflammatory or of a neoplastic nature, is responsible for the many studies which have appeared on the appendix vermiformis.

There are normal conditions, locations, sizes and general relations of the appendix. Any deviation from the normal we consider abnormal. How can the nature and the frequency of these abnormalities be determined, be they abnormalities in location, in size, in general relation, in anatomical integrity? By the comparison and the discussion of observations made in the dissecting room, on the operating table and in the post-mortem room. This paper is based exclusively on observations made in the latter. The post-mortem records of 3550 consecutive and unselected autopsies, held in the Cook County Hospital between January 1, 1893, and December 30, 1905, inclusive, were examined. These post-mortems were held on patients who died in the institution. An autopsy in this institution can only be held in the absence of protest from friends or relatives. No special space in the records was allowed to the appendix until the year 1896. Before that time the nature, the frequency and the importance of inflammations of this organ were not as fully understood and as fully appreciated as they are now.

The frequency of adhesion of the appendix vermiformis to neighboring structures and organs impressed us.

The appendix vermiformis was found adherent to neighboring structures or viscera 486 times. It was not possible to determine accurately in what proportion of cases the condition of "adherent appendix" was due to a previous inflammatory process of the appendix, or to a previous inflammatory process extending to the appendix from adjacent structures, in which it had originated. These adhesions are of interest to the clinician, to the pathologist and to the surgeon. They are frequently the cause of obscure (obscure as to correct interpretation) abdominal pains (adhesions to colon, to small intestines, to abdominal wall); of digestive disturbances (adhesions to stomach, liver, gall bladder). They may be the cause of vesical, of rectal tenesmus (adhesions to the urinary bladder, to the sigmoid flexure of the colon, to the rectum).

Adhesions can lead to kinking, to twisting, to obstruction of the appendix, to interference with its circulation, to impairment of its peristaltic action; can be the means of extension of an inflammatory process from the appendix to the structure or organ, to which the appendix is adherent; can make the appendix serve the office of a band over which a loop of intestines may become kinked, or beneath which a coil of gut may become looped. In either case intestinal obstruction or strangulation results. The appendix may lie concealed in a mass of adhesions.

In 145 cases of chronic adhesive appendicitis examined and analyzed at the Boston City Hospital, 118 showed no evidence of any abdominal condition to which adhesion could be referred, other than a prior inflammation of the appendix. Hence, they can be considered cases of primary chronic adhesive appendicitis. In 27 cases other sources for the adhesions could be ascertained (secondary chronic adhesive appendicitis), as salpingitis, hydrosalpinx, myoma of uterus; in 3 cases, carcinoma of uterus, with other pelvic structures; in 3 cases, disease of the gall bladder; in 2, tubercular peritonitis, etc.

These adhesions always prolong the operative intervention and may lead the surgeon to completely modify his technique in appendectomy. For instance, in those cases in which the appendix is so closely adherent to the wall of the cecum that it appears almost a part of it, and can not with safety be separated from it. In such cases the appendix may be split lengthwise, and its mucous membrane removed and ligated at its junction with the cecum, and the wound in the appendix sutured.

The analysis of 486 cases in which the appendix was adherent shows the following:

Appendix adherent to cecum in 357 cases.
Appendix adherent to psoas muscle in 44 cases.
Appendix adherent to hernial sac in 5 cases.
Appendix adherent to omentum in 16 cases.
Appendix adherent to small intestines in 18 cases.
Appendix adherent to ascending colon in 10 cases.
Appendix adherent to parietal wall in 18 cases.
Appendix adherent to brim of pelvis in 8 cases.
Appendix adherent to rectum in 2 cases.
Appendix adherent to sigmoid flexure in 1 case.
Appendix adherent to stomach in 1 case.
Appendix adherent to liver in 2 cases.
Appendix adherent to urinary bladder in 1 case.

In the cases in which it was adherent to the psoas muscle, in some the course of the appendix was parallel to the long axis of the psoas muscle; in some transverse to it; in others oblique.

In cases of adherent appendix not included in the above, the appendix was adherent to more than one structure.

Cunningham says: "The following locations of the appendix vermiformis have been considered normal by one or more observers: (1) Over the brim, into the pelvis; (2) upwards behind the cecum; (3) upwards and inwards towards the spleen. In these 3550 cases it is reported that the appendix was located partially or wholly in the true pelvis 155 times. This fact shows the utility of rectal and of vaginal examinations in suspected cases of appendicitis; it explains the frequency of pelvic abscesses in suppurative inflammations of the appendix vermiformis, and the rupturing of some of these abscesses into the uterus, into the rectum, into the urinary bladder, etc."

The appendix vermiformis was found in a hernial sac five times, cases 6182, 6924, 5245, 5344, and in case G. B., June 26, 1896. In each of these cases we were dealing with a right inguinal hernia. In one, the appendix was the only viscus present. In two others, a part of the cecum was present in the hernial sac with the appendix, and in the fourth, small intestines and the appendix vermiformis formed the contents of the hernial sac. All these hernia were irreducible, owing to the presence of adhesions. In one of these cases (5245) many concretions were found in the appendix.

The appendix was retroperitoneal in 12 cases. A retroperitoneal appendix is liable, if it becomes inflamed, to cause a retrocecal or retrocolic abscess. Retroperitoneal vermiform appendices play an important part in the causation of subphrenic abscesses.

Kelly and Hurdon say: "The question whether the appendix is an intraperitoneal or extraperitoneal organ is chiefly decided by the position it assumes in relation to the cecum or colon, whether it is downward or upward; or in more correct expression, early fusion between the colon and the posterior abdominal wall is apt to produce an ascending or retroperitoneal appendix, while late fusion brings about a pendant intraperitoneal appendix."

In case 5259, patient six months old, the appendix was found in the ascending colon. The lower end of the ileum had passed upward through the ileocecal valve with the cecum into the colon. The appendix at its attached end had been inverted with the bowel. The half below the constriction was gangrenous.

Case 6432. In this case the appendix was herniated through its own mesentery. It was not the seat of adhesions.

In case 5750, a case of gangrenous appendicitis, the cecal end of the appendix opened into a large abscess cavity in the liver. The opening in the appendix corresponded to the site of the liver abscess.

In twenty cases the appendix was partially or completely obliterated. It is said that total obliteration of the canal insures perfect immunity from further attacks, but that if any portion remains pervious there is an increased disposition to other attacks. In nine of these cases the obliteration was due to an inflammation; in the others it was not determined whether the obliteration was inflammatory or involutionary in nature. In one case constriction was proximal to a concretion; in another the appendix contained mucoid material distal to the constriction; in one the end distal to the constriction contained pus.

The appendix was found kinked in twenty cases. Some of these bends and kinks were due to inflammatory adhesions; some were due to a shortened meso-appendix. In ten of these cases the kinking was inflammatory in origin; in the others, reports are too meager to state the causes. Constrictions of the appendix were noticed in nine cases. They were all due to previous inflammation of the organ.

In these reports no case of supernumerary appendix is recorded. No case of absence of the appendix not attributable to appendectomy or sloughing was seen. The absence due to operation of the appendix was noted twenty-one times.

The size of the appendix is more variable than its position. Kelly and Hurdon agree with Ribbert, Berry and others in placing the average length of the appendix at about 8.3 cm., or between three and three and one-half inches. Schlange, in von Bergmann's Practice of Surgery, gives the average length as 9.2 cm., equal to three and one-half inches.

In case 6656, the length of the appendix was ½ cm.

In case 6791, the length of the appendix was 3 cm.

In case 7073, the length of the appendix was 3 cm.

In case 6107, the length of the appendix was 21 cm.

In case 5616, the appendix was normal, but was nine inches in length.

In one case (patient, Isaac Williams, posted April 26, 1895), the appendix is recorded as having been ten inches long. Long appendices are frequently bent upon themselves or drawn up by the shortness of their mesentery into various bizarre forms, figure-of-eight, or spiral.

Foreign bodies were found in the appendix vermiformis as follows:

Grape seed, one case.

Fish bone (1½ inches in length), covered with concretions, one case.

Enteroliths, twenty-five cases.

By enteroliths we understand fecal material which has undergone desiccation. The ordinary or normal appendix may contain fecal material similar to that found in the adjacent large intestine. Bryant found fecal matter in 70 per cent of his adult specimens. In some of the cases reported above the enteroliths were single, in others multiple. In case 5030 there were two large and several small concretions.

In these 3550 autopsies the appendix vermiformis was reported to have been the seat of tubercular lesions ten times: cases 5507, 6701, 5275, 5421, 6499, 6504, 6779, 6225, 5104, 5982. A fact worthy of note is that in each and every one of these ten cases the tubercular lesions in the appendix coexisted with tubercular lesions elsewhere in the organism; that in all of these cases tuberculous pneumonitis of one or other variety was invariably present. Not one of these cases of tuberculous appendicitis was primary. These were all secondary, either by continuity of tissue, as extension from tuberculosis of neighboring coils of intestine; or by vascular transplantation. We are forced to state that tuberculosis of the appendix vermiformis is but exceptionally primary and isolated. In four of these cases the organ was free, was non-adherent; in six it was adherent to some neighboring structure. In some of these cases the tuberculous process in the appendix vermiformis had led to the formation of caseous areas; in others to ulcer formation; in others simply to the formation of tuberculous granulation tissue. In some the process was limited to the internal coats; in others to the external coats; in others it involved all the coats. In all these cases the tuberculous appendicitis was not productive of symptoms sufficiently marked to lead to its diagnosis during life.

Twice (cases 5305, 5912) the appendix was the seat of typhoidal disease. In both of these cases typhoidal lesions in other parts of the abdomen coexisted (intestines, mesenteric glands, spleen). In case 5305 there were submucous hemorrhages; in case 5912 ulcers were present.

In case 5272 there was a cavity between the folds of the meso-appendix communicating with the lumen of the appendix, and containing thick pus.

The appendix was found to be the seat of acute inflammation (non-suppurative in character, that had not been pus-producing) forty-one times. In six cases pus was found in the cavity of the appendix, that is, in six cases we had an empyema of the appendix.

The appendix vermiformis was found to be the seat of neoplastic disease three times (cases 6200, 6178, 6002). In each of these cases the neoplasm was a carcinoma. In each of these cases the appendix had been involved secondarily by the neoplastic process. In each the primary tumor was in the stomach. Benign neoplastic or sarçomatous change in the appendix was not found in any case. In two of the cases reported the tumor was apparently secondary by vascular transplantation; in one secondary by extension by contiguity. (Appendix was adherent to stomach by tumor mass.)

The following shows the great improvement in the understanding of indications for operation in appendicitis, and in the performance of the various operations for this condition that has taken place during the last decade:

Between the years 1893 and 1896 there came to the autopsy table at the Cook County Hospital nineteen cases, which had been operated on for appendicitis, and in which suppurative peritonitis was present; while between the years 1896 and 1905, inclusive, there only came to the autopsy table five such cases.

The operation performed in those days is best understood and appreciated by the following, taken from the post-mortem records:

Case of L. Jackson, March 20, 1895.—In right iliac region wound is found 7 cm. long, partly closed by sutures. Through this incision protrudes a loop of intestine and a gauze drain; appendix was found adherent to psoas, and had a perforation at lower third. (Operation for appendicitis.)

Case of L. Jones, examined February 10, 1895.—In abdominal wall in median line an incision of about four inches in length was found packed with iodoform gauze. Omentum and intestines found matted together. Appendix found bound down to psoas and red in appearance. Constriction about three-fourths cm. from tip. (General suppurative peritonitis.)

Case of J. G. Simons, February 4, 1893.—Appendix, colon and omentum found adherent in right iliac region. Appendix surrounded by granulating tissue. Looks, on separating adhesions, like a large ulcerating cavity.

Case of Joseph Kubat, April 7, 1895.—In right lower quadrant, eight-inch scar is found; omentum adherent to peritoneum under scar. Appendix present and adherent to abdominal wall.

These post-mortem records affirm the following facts concerning the appendix vermiformis:

1. That it is almost always an intraperitoneal organ; exceptionally, it is extraperitoneal, and then usually only partly so.

2. That it has been found in nearly every portion of the abdominal or pelvic cavities.

3. That it may form the contents or part of the contents of a hernial sac.

4. That its presence in a hernial sac does not render it immune from the lesions to which it is subject when normally located.

5. That it may be adherent to any intraperitoneal organ or structure.

6. That it may be adherent to some extraperitoneal structures, kidney, retrocolic cellular tissues, etc.

7. That pathological conditions have been found which seem to indicate that inflammations can extend from it to neighboring organs and structures to which it is adherent, and vice versa.

8. That in the diagnosing of obscure abdominal and pelvic conditions the probability of a previous or of an existing appendicitis must be considered.

9. That pus may be present within the cavity of the appendix, within the walls of the appendix, or the condition of peri-appendiceal abscess may occur.

10. That inflammations of the appendix may terminate in resolution, in adhesion formation, in obliteration of the appendix (partial or complete), in interstitial thickening, in gangrene, ulceration and perforation of the organ; may terminate in suppuration.

11. That one attack of appendicitis predisposes to other attacks, until complete obliteration of the lumen of the appendix has taken place.

12. That the condition of supernumerary appendix does not occur.

13. That congenital absence of the appendix, if it occurs, is so infrequent as to be ignored, from a clinical standpoint.

14. That the appendix may vary in length from ½ cm. to 26 cm.

15. That the lodgment of foreign bodies in the lumen of the appendix is an infrequent occurrence, only two cases, excluding enteroliths, having been observed in 3560 cases.

16. That neoplastic disease of the appendix is uncommon. We are inclined to think that neoplasms of the appendix are almost always secondary either by continuity or contiguity of tissue, or by vascular transplantation. We have never met with a primary case. Some primary cases, however, have been reported.

17. That this organ may be the seat of lesions of the same nature as can occur in other portions of the alimentary canal, viz.: typhoidal, tubercular, actinomycotic, dysenteric, etc.

18. That tuberculous appendicitis is almost invariably secondary.

19. That the lessened frequency during the last decade of diffuse suppurative peritonitis following operations for appendicitis is due, first, to more exact diagnosis; second, to earlier operation; third, to excision of the appendix and of its mesentery in cases not complicated by periappendiceal abscess; fourth, to better and more perfect technique on part of operator.

20. To limiting the surgical intervention in cases of periappendiceal abscess to incision and evacuation and drainage of the pus cavity, if the appendix be not easily accessible.

